# Methyl 3-hydr­oxy-4-oxo-3,4-dihydro-2*H*-1,2-benzothia­zine-3-carboxyl­ate 1,1-dioxide monohydrate

**DOI:** 10.1107/S1600536808030948

**Published:** 2008-09-30

**Authors:** Muhammad Nadeem Arshad, M. Nawaz Tahir, Islam Ullah Khan, Muhammad Shafiq, Waseeq Ahmad Siddiqui

**Affiliations:** a Department of Chemistry, Government College University, Lahore, Pakistan; b Department of Physics, University of Sargodha, Sargodha, Pakistan; c Department of Chemistry, University of Sargodha, Sargodha, Pakistan

## Abstract

In the mol­ecule of the title compound, C_10_H_9_NO_6_S·H_2_O, the benzothia­zine ring adopts an envelope conformation. An intra­molecular N—H⋯O hydrogen bond results in the formation of a nonplanar five-membered ring which has a twisted conformation. In the crystal structure, inter­molecular N—H⋯O, O—H⋯O and C—H⋯O hydrogen bonds link the mol­ecules to form a three-dimensional network. There is a π–π contact between the benzene rings [centroid–centroid distance = 3.972 (2) Å].

## Related literature

For general background, see: Shafiq, Khan *et al.* (2008 ▶); Shafiq, Tahir *et al.* (2008 ▶); Tahir *et al.* (2008 ▶). For related literature, see: Antsyshkina *et al.* (2003 ▶); Allen (2002 ▶). For bond-length data, see: Allen *et al.* (1987 ▶). For ring puckering parameters, see: Cremer & Pople (1975 ▶).
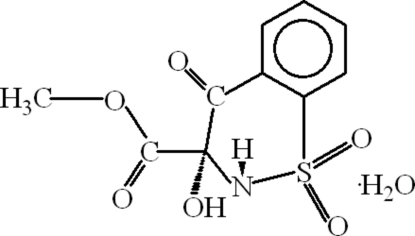


## Experimental

### 

#### Crystal data

C_10_H_9_NO_6_S·H_2_O
*M* *_r_* = 289.26
Orthorhombic, 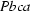

*a* = 7.7504 (5) Å
*b* = 14.5638 (9) Å
*c* = 21.0615 (14) Å
*V* = 2377.3 (3) Å^3^
*Z* = 8
Mo *K*α radiation
μ = 0.30 mm^−1^
*T* = 296 (2) K
0.24 × 0.18 × 0.15 mm

#### Data collection

Bruker Kappa APEXII CCD diffractometer
Absorption correction: multi-scan (*SADABS*; Bruker, 2005 ▶) *T*_min_ = 0.934, *T*_max_ = 0.958
14889 measured reflections
2998 independent reflections
1895 reflections with *I* > 2σ(*I*)
*R*_int_ = 0.065

#### Refinement

*R*[*F* ^2^ > 2σ(*F* ^2^)] = 0.047
*wR*(*F* ^2^) = 0.122
*S* = 1.01
2998 reflections
184 parameters
H atoms treated by a mixture of independent and constrained refinement
Δρ_max_ = 0.40 e Å^−3^
Δρ_min_ = −0.32 e Å^−3^

### 

Data collection: *APEX2* (Bruker, 2007 ▶); cell refinement: *APEX2*; data reduction: *SAINT* (Bruker, 2007 ▶); program(s) used to solve structure: *SHELXS97* (Sheldrick, 2008 ▶); program(s) used to refine structure: *SHELXL97* (Sheldrick, 2008 ▶); molecular graphics: *ORTEP-3 for Windows* (Farrugia, 1997 ▶) and *PLATON* (Spek, 2003 ▶); software used to prepare material for publication: *WinGX* (Farrugia, 1999 ▶) and *PLATON*.

## Supplementary Material

Crystal structure: contains datablocks global, I. DOI: 10.1107/S1600536808030948/hk2537sup1.cif

Structure factors: contains datablocks I. DOI: 10.1107/S1600536808030948/hk2537Isup2.hkl

Additional supplementary materials:  crystallographic information; 3D view; checkCIF report

## Figures and Tables

**Table 1:** Hydrogen-bond geometry (Å, °)

*D*—H⋯*A*	*D*—H	H⋯*A*	*D*⋯*A*	*D*—H⋯*A*
N1—H1⋯O6	0.78 (3)	2.43 (3)	2.744 (3)	106 (2)
N1—H1⋯O7^i^	0.78 (3)	2.29 (3)	3.032 (3)	162 (3)
O4—H4*O*⋯O7^ii^	0.84 (3)	1.94 (3)	2.773 (3)	175 (2)
O7—H71⋯O3^iii^	0.83 (3)	2.45 (3)	3.107 (3)	137 (3)
O7—H71⋯O5^iv^	0.83 (3)	2.45 (3)	3.028 (3)	128 (3)
O7—H72⋯O2	0.83 (4)	2.21 (4)	3.027 (3)	167 (3)
C5—H5⋯O4^iv^	0.9300	2.4800	3.374 (3)	162.00
C10—H10*A*⋯O4^i^	0.9600	2.3100	2.994 (3)	128.00

Symmetry codes: (i) 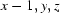; (ii) 
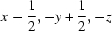
; (iii) 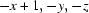; (iv) 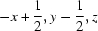.

**Fig. 1.**
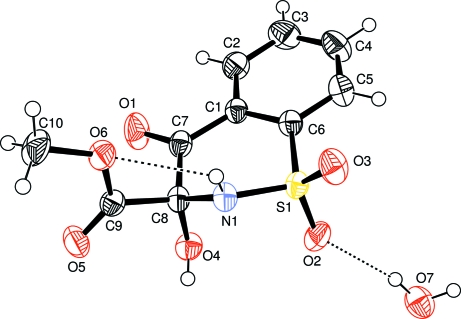
The molecular structure of the title molecule, with the atom-numbering scheme. Displacement ellipsoids are drawn at the 50% probability level. Hydrogen bonds are shown as dotted lines.

**Fig. 2.**
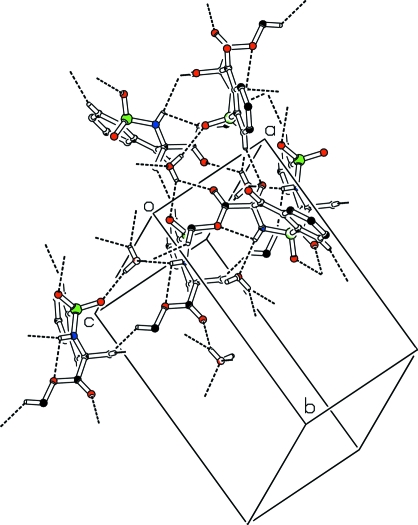
A packing diagram of the title compound. Hydrogen bonds are shown as dashed lines.

## supplementary crystallographic information

### Comment

The title compound has been prepared in continuation of research on benzo- thiazine derivatives (Shafiq, Khan *et al.*, 2008; Shafiq, Tahir *et al.*, 2008; Tahir *et al.*,  2008) by our research group. The CCDC search (Allen, 2002) shows that a single crystal structure has been reported, in which the same benzothiazine ring exists (Antsyshkina *et al.*,  2003). The title compound differs from the reported structure, due to the hydroxy and methylformate groups. Due to the hydroxy group, the S-configuration in the title compound has been confirmed.

In the molecule of the title compound (Fig. 1), the bond lengths (Allen *et al.*,  1987) and angles are within normal ranges. Ring A (C1-C6) is, of course, planar. Ring B (S1/N1/C1/C6-C8) is not planar, having total puckering amplitude, Q_T_, of 0.733 (3) Å and envelope conformation [φ = 21.21 (3)° and θ = 76.70 (3)°] (Cremer & Pople, 1975) with N1 atom displaced by 0.575 (3) Å from the plane of the other ring atoms. The intramolecular N-H···O hydrogen bond (Table 1) results in the formation of a nonplanar five-membered ring C (N1/O6/C8/C9/H1), having twisted conformation.

In the crystal structure, intermolecular N-H···O, O-H···O and C-H···O hydrogen bonds (Table 1) link the molecules to form a three dimensional network (Fig. 2), in which they may be effective in the stabilization of the structure. The π—π contact between the benzene rings, Cg2···Cg2^i^ [symmetry code: (i) -1/2 + x, y, 1/2 - z, where Cg2 is the centroid of the ring A (C1-C6)] may further stabilize the structure, with centroid-centroid distance of 3.972 (2) Å.

### Experimental

For the preparation of the title compound, methyl 4-hydroxy-2*H*-1,2 -benzothiazine-3-carboxylate 1,1-dioxide (0.5 g, 1.95 mmol), *N*-bromo- succinamide (0.38 g, 2.145 mmol) and dibenzoyl peroxide (0.035 g, 0.15 mmol) were added in CCl_4_ (10 ml). The mixture was refluxed for 2 h. After the completion of reaction, CCl_4_ was distilled off under vacuum. The obtained residue was washed with hot water to remove other impurities. The solid product was recrystallized in water and methanol to obtain the suitable crystals for x-ray analysis.

### Refinement

H atoms were located in difference syntheses and refined as [O-H = 0.84 (3) Å (for OH); O-H = 0.83 (3) and 0.83 (4) Å (for H_2_O); N-H = 0.78 (3) Å (for NH). The remaining H atoms were positioned geometrically, with C-H = 0.93 and 0.96 Å for aromatic and methyl H, respectively, and constrained to ride on their parent atoms with U_iso_(H) = xU_eq_(C,N,O), where x = 1.5 for methyl H and x = 1.2 for all other H atoms.

### Crystal data

**Table:**

C_10_H_9_NO_6_S·H_2_O	*F*(000) = 1200
*M**_r_* = 289.26	*D*_x_ = 1.616 Mg m^−^^3^
Orthorhombic, *P**b**c**a*	Mo *K*α radiation, λ = 0.71073 Å
Hall symbol: -P 2ac 2ab	Cell parameters from 2998 reflections
*a* = 7.7504 (5) Å	θ = 3.0–28.5°
*b* = 14.5638 (9) Å	µ = 0.30 mm^−^^1^
*c* = 21.0615 (14) Å	*T* = 296 K
*V* = 2377.3 (3) Å^3^	Prismatic, colourless
*Z* = 8	0.24 × 0.18 × 0.15 mm

### Data collection

**Table:**

Bruker Kappa APEXII CCD diffractometer	2998 independent reflections
Radiation source: fine-focus sealed tube	1895 reflections with *I* > 2σ(*I*)
graphite	*R*_int_ = 0.065
Detector resolution: 7.40 pixels mm^-1^	θ_max_ = 28.5°, θ_min_ = 3.0°
ω scans	*h* = −10→10
Absorption correction: multi-scan (SADABS; Bruker, 2005)	*k* = −19→10
*T*_min_ = 0.934, *T*_max_ = 0.958	*l* = −28→27
14889 measured reflections	

### Refinement

**Table:**

Refinement on *F*^2^	Primary atom site location: structure-invariant direct methods
Least-squares matrix: full	Secondary atom site location: difference Fourier map
*R*[*F*^2^ > 2σ(*F*^2^)] = 0.047	Hydrogen site location: inferred from neighbouring sites
*wR*(*F*^2^) = 0.122	H atoms treated by a mixture of independent and constrained refinement
*S* = 1.01	*w* = 1/[σ^2^(*F*_o_^2^) + (0.0491*P*)^2^ + 1.1022*P*] where *P* = (*F*_o_^2^ + 2*F*_c_^2^)/3
2998 reflections	(Δ/σ)_max_ < 0.001
184 parameters	Δρ_max_ = 0.40 e Å^−^^3^
0 restraints	Δρ_min_ = −0.32 e Å^−^^3^

### Special details

**Table:**

Geometry. Bond distances, angles *etc*. have been calculated using the rounded fractional coordinates. All su's are estimated from the variances of the (full) variance-covariance matrix. The cell e.s.d.'s are taken into account in the estimation of distances, angles and torsion angles
Refinement. Refinement of *F*^2^ against ALL reflections. The weighted *R*-factor *wR* and goodness of fit *S* are based on *F*^2^, conventional *R*-factors *R* are based on *F*, with *F* set to zero for negative *F*^2^. The threshold expression of *F*^2^ > σ(*F*^2^) is used only for calculating *R*-factors(gt) *etc*. and is not relevant to the choice of reflections for refinement. *R*-factors based on *F*^2^ are statistically about twice as large as those based on *F*, and *R*- factors based on ALL data will be even larger.

### Fractional atomic coordinates and isotropic or equivalent isotropic displacement parameters (Å^2^)

**Table:**

	*x*	*y*	*z*	*U*_iso_*/*U*_eq_	
S1	0.18084 (8)	0.12546 (4)	0.08206 (3)	0.0314 (2)	
O1	0.0037 (3)	0.32676 (13)	0.21865 (9)	0.0568 (8)	
O2	0.3475 (2)	0.15746 (13)	0.06338 (9)	0.0450 (7)	
O3	0.1137 (3)	0.04539 (12)	0.05197 (9)	0.0450 (6)	
O4	0.2024 (2)	0.33701 (12)	0.09947 (10)	0.0360 (6)	
H4O	0.204 (4)	0.3501 (19)	0.0608 (14)	0.0432*	
O5	−0.0985 (3)	0.42862 (12)	0.08273 (10)	0.0460 (7)	
O6	−0.2498 (2)	0.30222 (13)	0.10613 (9)	0.0413 (6)	
O7	0.7145 (3)	0.10998 (15)	0.02689 (11)	0.0481 (7)	
H71	0.731 (4)	0.054 (2)	0.0226 (15)	0.0577*	
H72	0.615 (5)	0.116 (2)	0.0412 (16)	0.0577*	
N1	0.0398 (3)	0.20645 (14)	0.07099 (10)	0.0308 (6)	
H1	−0.052 (4)	0.1857 (18)	0.0675 (13)	0.0370*	
C1	0.1243 (3)	0.17959 (16)	0.20533 (12)	0.0298 (7)	
C2	0.1294 (4)	0.16328 (19)	0.27020 (13)	0.0414 (9)	
H2	0.09091	0.20830	0.29812	0.0497*	
C3	0.1908 (4)	0.0814 (2)	0.29384 (14)	0.0518 (10)	
H3	0.19348	0.07164	0.33746	0.0623*	
C4	0.2479 (5)	0.0141 (2)	0.25324 (15)	0.0525 (10)	
H4	0.28955	−0.04093	0.26959	0.0629*	
C5	0.2440 (4)	0.02761 (18)	0.18834 (14)	0.0444 (9)	
H5	0.28239	−0.01804	0.16088	0.0533*	
C6	0.1824 (3)	0.10985 (16)	0.16477 (12)	0.0306 (7)	
C7	0.0552 (3)	0.26875 (16)	0.18234 (12)	0.0319 (8)	
C8	0.0514 (3)	0.28972 (15)	0.11076 (11)	0.0284 (7)	
C9	−0.1075 (3)	0.35000 (17)	0.09736 (12)	0.0312 (8)	
C10	−0.4122 (3)	0.3507 (2)	0.10026 (15)	0.0500 (10)	
H10A	−0.50577	0.30900	0.10785	0.0750*	
H10B	−0.42207	0.37576	0.05825	0.0750*	
H10C	−0.41623	0.39963	0.13080	0.0750*	

### Atomic displacement parameters (Å^2^)

**Table:**

	*U*^11^	*U*^22^	*U*^33^	*U*^12^	*U*^13^	*U*^23^
S1	0.0315 (3)	0.0292 (3)	0.0334 (3)	0.0025 (3)	0.0002 (3)	−0.0061 (3)
O1	0.0826 (16)	0.0444 (11)	0.0433 (12)	0.0237 (12)	−0.0002 (11)	−0.0134 (9)
O2	0.0328 (11)	0.0479 (11)	0.0544 (12)	0.0054 (9)	0.0128 (9)	−0.0020 (9)
O3	0.0591 (13)	0.0327 (9)	0.0433 (11)	0.0012 (9)	−0.0078 (10)	−0.0121 (8)
O4	0.0240 (9)	0.0376 (10)	0.0463 (11)	−0.0060 (8)	−0.0014 (8)	−0.0029 (9)
O5	0.0406 (12)	0.0312 (10)	0.0662 (14)	0.0041 (8)	−0.0023 (10)	0.0090 (9)
O6	0.0220 (9)	0.0402 (10)	0.0618 (13)	−0.0003 (8)	0.0007 (9)	0.0062 (9)
O7	0.0453 (13)	0.0400 (10)	0.0590 (14)	−0.0025 (10)	−0.0070 (10)	0.0005 (10)
N1	0.0258 (11)	0.0296 (10)	0.0370 (12)	−0.0008 (9)	−0.0049 (10)	−0.0052 (9)
C1	0.0261 (12)	0.0291 (12)	0.0342 (13)	0.0004 (10)	−0.0018 (10)	−0.0021 (10)
C2	0.0470 (16)	0.0411 (15)	0.0362 (14)	0.0006 (13)	0.0007 (13)	−0.0049 (12)
C3	0.063 (2)	0.0559 (18)	0.0364 (15)	0.0002 (17)	−0.0064 (15)	0.0089 (14)
C4	0.067 (2)	0.0393 (15)	0.0513 (18)	0.0054 (15)	−0.0086 (16)	0.0110 (14)
C5	0.0503 (17)	0.0342 (14)	0.0487 (17)	0.0069 (13)	−0.0052 (14)	−0.0021 (13)
C6	0.0269 (12)	0.0317 (12)	0.0332 (13)	0.0008 (11)	−0.0037 (11)	−0.0016 (10)
C7	0.0284 (13)	0.0296 (12)	0.0377 (14)	0.0005 (10)	−0.0013 (11)	−0.0055 (11)
C8	0.0240 (12)	0.0257 (11)	0.0356 (14)	−0.0001 (10)	−0.0027 (10)	−0.0041 (10)
C9	0.0272 (13)	0.0331 (13)	0.0332 (14)	−0.0001 (11)	−0.0003 (10)	−0.0017 (10)
C10	0.0234 (14)	0.0674 (19)	0.0591 (19)	0.0118 (14)	−0.0015 (13)	0.0030 (16)

### Geometric parameters (Å, °)

**Table:**

S1—O2	1.4284 (17)	C1—C7	1.486 (3)
S1—O3	1.426 (2)	C1—C6	1.402 (3)
S1—N1	1.625 (2)	C2—C3	1.377 (4)
S1—C6	1.757 (3)	C3—C4	1.374 (4)
O1—C7	1.207 (3)	C4—C5	1.381 (4)
O4—C8	1.379 (3)	C5—C6	1.382 (4)
O5—C9	1.188 (3)	C7—C8	1.539 (3)
O6—C9	1.317 (3)	C8—C9	1.539 (3)
O6—C10	1.449 (3)	C2—H2	0.9300
O4—H4O	0.84 (3)	C3—H3	0.9300
O7—H71	0.83 (3)	C4—H4	0.9300
O7—H72	0.83 (4)	C5—H5	0.9300
N1—C8	1.477 (3)	C10—H10C	0.9600
N1—H1	0.78 (3)	C10—H10A	0.9600
C1—C2	1.387 (4)	C10—H10B	0.9600
			
S1···H10B^i^	3.0600	N1···O6	2.744 (3)
O1···O4	2.949 (3)	N1···O7^xii^	3.032 (3)
O1···O6	3.099 (3)	C3···C5^x^	3.570 (4)
O1···C4^ii^	3.406 (4)	C4···O1^ix^	3.418 (4)
O1···C4^iii^	3.418 (4)	C4···O1^xiii^	3.405 (4)
O2···O4	2.946 (3)	C5···C3^xi^	3.570 (4)
O2···O7	3.027 (3)	C5···O4^ix^	3.374 (3)
O2···C9^i^	3.405 (3)	C9···O2^vii^	3.405 (3)
O3···C10^iv^	3.393 (3)	C10···O4^xii^	2.994 (3)
O3···O7^v^	3.107 (3)	C10···O3^xiv^	3.393 (3)
O3···O3^vi^	3.106 (3)	C10···H4O^xii^	3.09 (3)
O4···O5	2.710 (3)	C10···H2^x^	2.9800
O4···C5^iii^	3.374 (3)	H1···O6	2.43 (3)
O4···O7^vii^	2.773 (3)	H1···O7^xii^	2.29 (3)
O4···O2	2.946 (3)	H2···H10A^xi^	2.5800
O4···O1	2.949 (3)	H2···O1	2.5000
O4···C10^viii^	2.994 (3)	H2···O6^xi^	2.7300
O5···O7^iii^	3.028 (3)	H2···C10^xi^	2.9800
O5···O4	2.710 (3)	H3···O7^x^	2.9200
O6···N1	2.744 (3)	H3···O5^xiii^	2.7800
O6···O1	3.099 (3)	H4···H10C^xiii^	2.4700
O7···N1^viii^	3.032 (3)	H4···O1^ix^	2.7300
O7···O5^ix^	3.028 (3)	H4O···C10^viii^	3.09 (3)
O7···O4^i^	2.773 (3)	H4O···H10A^viii^	2.5300
O7···O3^v^	3.107 (3)	H4O···O5	2.65 (3)
O7···O2	3.027 (3)	H4O···O7^vii^	1.94 (3)
O1···H4^iii^	2.7300	H4O···H71^vii^	2.25 (4)
O1···H2	2.5000	H4O···H72^vii^	2.31 (4)
O2···H72	2.21 (4)	H5···O3	2.8000
O2···H10A^viii^	2.6500	H5···O4^ix^	2.4800
O3···H10B^i^	2.6000	H10A···O2^xii^	2.6500
O3···H5	2.8000	H10A···O4^xii^	2.3100
O3···H71^v^	2.45 (3)	H10A···H2^x^	2.5800
O3···H10B^iv^	2.8900	H10A···H4O^xii^	2.5300
O4···H5^iii^	2.4800	H10B···S1^vii^	3.0600
O4···H10A^viii^	2.3100	H10B···O3^vii^	2.6000
O5···H4O	2.65 (3)	H10B···O5	2.6700
O5···H10B	2.6700	H10B···O3^xiv^	2.8900
O5···H3^ii^	2.7800	H10C···O5	2.7000
O5···H72^iii^	2.87 (3)	H10C···H4^ii^	2.4700
O5···H10C	2.7000	H71···H4O^i^	2.25 (4)
O5···H71^iii^	2.45 (3)	H71···O3^v^	2.45 (3)
O6···H2^x^	2.7300	H71···O5^ix^	2.45 (3)
O6···H1	2.43 (3)	H72···O2	2.21 (4)
O7···H1^viii^	2.29 (3)	H72···H4O^i^	2.31 (5)
O7···H4O^i^	1.94 (3)	H72···O5^ix^	2.87 (3)
O7···H3^xi^	2.9200		
			
O2—S1—O3	118.33 (12)	O1—C7—C8	118.4 (2)
O2—S1—N1	109.39 (11)	O4—C8—N1	111.36 (19)
O2—S1—C6	108.01 (11)	O4—C8—C7	104.59 (19)
O3—S1—N1	106.53 (12)	O4—C8—C9	111.27 (18)
O3—S1—C6	109.72 (11)	N1—C8—C7	113.20 (18)
N1—S1—C6	103.94 (11)	N1—C8—C9	108.41 (19)
C9—O6—C10	117.3 (2)	C7—C8—C9	107.96 (19)
C8—O4—H4O	107 (2)	O5—C9—C8	123.4 (2)
H71—O7—H72	107 (3)	O6—C9—C8	110.1 (2)
S1—N1—C8	118.28 (17)	O5—C9—O6	126.5 (2)
C8—N1—H1	115 (2)	C3—C2—H2	120.00
S1—N1—H1	110 (2)	C1—C2—H2	120.00
C2—C1—C6	117.8 (2)	C2—C3—H3	120.00
C2—C1—C7	118.7 (2)	C4—C3—H3	120.00
C6—C1—C7	123.4 (2)	C5—C4—H4	120.00
C1—C2—C3	120.9 (3)	C3—C4—H4	120.00
C2—C3—C4	120.3 (3)	C4—C5—H5	120.00
C3—C4—C5	120.5 (3)	C6—C5—H5	120.00
C4—C5—C6	119.1 (3)	O6—C10—H10B	109.00
S1—C6—C5	118.1 (2)	O6—C10—H10C	109.00
S1—C6—C1	120.56 (18)	O6—C10—H10A	110.00
C1—C6—C5	121.4 (2)	H10A—C10—H10C	109.00
O1—C7—C1	121.6 (2)	H10B—C10—H10C	109.00
C1—C7—C8	120.0 (2)	H10A—C10—H10B	110.00
			
O2—S1—N1—C8	−67.0 (2)	C2—C1—C7—C8	178.7 (2)
O3—S1—N1—C8	163.98 (18)	C6—C1—C7—O1	178.8 (2)
C6—S1—N1—C8	48.1 (2)	C6—C1—C7—C8	−2.4 (3)
O2—S1—C6—C1	94.5 (2)	C1—C2—C3—C4	−0.2 (5)
O2—S1—C6—C5	−84.8 (2)	C2—C3—C4—C5	−0.2 (5)
O3—S1—C6—C1	−135.2 (2)	C3—C4—C5—C6	0.2 (5)
O3—S1—C6—C5	45.5 (2)	C4—C5—C6—S1	179.4 (2)
N1—S1—C6—C1	−21.6 (2)	C4—C5—C6—C1	0.1 (4)
N1—S1—C6—C5	159.1 (2)	O1—C7—C8—O4	84.6 (3)
C10—O6—C9—O5	−3.0 (4)	O1—C7—C8—N1	−154.0 (2)
C10—O6—C9—C8	175.4 (2)	O1—C7—C8—C9	−33.9 (3)
S1—N1—C8—O4	64.3 (2)	C1—C7—C8—O4	−94.2 (2)
S1—N1—C8—C7	−53.3 (3)	C1—C7—C8—N1	27.2 (3)
S1—N1—C8—C9	−173.01 (16)	C1—C7—C8—C9	147.2 (2)
C6—C1—C2—C3	0.5 (4)	O4—C8—C9—O5	−4.0 (3)
C7—C1—C2—C3	179.4 (3)	O4—C8—C9—O6	177.6 (2)
C2—C1—C6—S1	−179.7 (2)	N1—C8—C9—O5	−126.8 (3)
C2—C1—C6—C5	−0.4 (4)	N1—C8—C9—O6	54.8 (3)
C7—C1—C6—S1	1.4 (3)	C7—C8—C9—O5	110.2 (3)
C7—C1—C6—C5	−179.3 (2)	C7—C8—C9—O6	−68.2 (2)
C2—C1—C7—O1	−0.1 (4)		

Symmetry codes: (i) *x*+1/2, −*y*+1/2, −*z*; (ii) −*x*, *y*+1/2, −*z*+1/2; (iii) −*x*+1/2, *y*+1/2, *z*; (iv) −*x*−1/2, *y*−1/2, *z*; (v) −*x*+1, −*y*, −*z*; (vi) −*x*, −*y*, −*z*; (vii) *x*−1/2, −*y*+1/2, −*z*; (viii) *x*+1, *y*, *z*; (ix) −*x*+1/2, *y*−1/2, *z*; (x) *x*−1/2, *y*, −*z*+1/2; (xi) *x*+1/2, *y*, −*z*+1/2; (xii) *x*−1, *y*, *z*; (xiii) −*x*, *y*−1/2, −*z*+1/2; (xiv) −*x*−1/2, *y*+1/2, *z*.

### Hydrogen-bond geometry (Å, °)

**Table:**

*D*—H···*A*	*D*—H	H···*A*	*D*···*A*	*D*—H···*A*
N1—H1···O6	0.78 (3)	2.43 (3)	2.744 (3)	106 (2)
N1—H1···O7^xii^	0.78 (3)	2.29 (3)	3.032 (3)	162 (3)
O4—H4O···O7^vii^	0.84 (3)	1.94 (3)	2.773 (3)	175 (2)
O7—H71···O3^v^	0.83 (3)	2.45 (3)	3.107 (3)	137 (3)
O7—H71···O5^ix^	0.83 (3)	2.45 (3)	3.028 (3)	128 (3)
O7—H72···O2	0.83 (4)	2.21 (4)	3.027 (3)	167 (3)
C5—H5···O4^ix^	0.9300	2.4800	3.374 (3)	162.00
C10—H10A···O4^xii^	0.9600	2.3100	2.994 (3)	128.00

Symmetry codes: (xii) *x*−1, *y*, *z*; (vii) *x*−1/2, −*y*+1/2, −*z*; (v) −*x*+1, −*y*, −*z*; (ix) −*x*+1/2, *y*−1/2, *z*.
